# Back Problems: Pros and Cons of Core Strengthening Exercises as a Part of Athlete Training

**DOI:** 10.3390/ijerph18105400

**Published:** 2021-05-18

**Authors:** Erika Zemková, Ludmila Zapletalová

**Affiliations:** 1 Department of Biological and Medical Sciences, Faculty of Physical Education and Sports, Comenius University in Bratislava, 814 69 Bratislava, Slovakia; 2 Sports Technology Institute, Faculty of Electrical Engineering and Information Technology, Slovak University of Technology, 812 19 Bratislava, Slovakia; 3 Faculty of Health Sciences, University of Ss. Cyril and Methodius in Trnava, 917 01 Trnava, Slovakia; zapletalovalidi@gmail.com

**Keywords:** athletes, back pain, core strength and endurance, exercise load

## Abstract

While competitive training is usually associated with the prevalence of back pain and injuries in athletes, little attention is being paid to the positive effects of sport-specific exercises on core musculature in the prevention of back problems. This scoping review aims (i) to map the literature that addresses the effects on reduction of back problems following athlete training with differing demands on the core musculature and (ii) to identify gaps in the existing literature and propose future research on this topic. The main literature search was conducted on the MEDLINE, PubMed, Web of Science, Scopus, and Cochrane Library databases and was completed on Elsevier, SpringerLink, and Google Scholar. A total of 21 research articles met the inclusion criteria. The findings of 17 studies identified that core strengthening and core stabilization exercises, alone or in combination with athlete training, contribute to the reduction of back pain in athletes, whereas only four studies revealed no significant association of core muscle strength and/or endurance with back problems. Nevertheless, more research is warranted to elucidate the pros and cons of purely sport-specific training with differing demands on the core musculature on back health in athletes. This could help us to design prevention strategies specifically tailored to individual athletes.

## 1. Introduction

Back pain is the most common health problem in athletes that may affect their performance [1]. In particular, low back pain (LBP) is among the most prevalent musculoskeletal conditions with incidence rates of 1–30% [2]. The LBP prevalence is 1–94% (the highest in rowing and cross-country skiing), while the point prevalence is 18–65% (the highest in rowing and the lowest in basketball) [1]. The highest prevalence rates are during the peak season [3].

There are many potential risk factors for back pain in athletes. However, evidence exists only for previous low back pain, decreased lumbar extension or flexion, and high body weight [4]. The strength of the relationship between risk factors and back pain depends on the type of sport, level of competition, and training characteristics [4]. Lifting heavy weights, falling, being tackled in team sports, or fighting in combat sports can cause back pain or injury [5,6,7,8]. However, when the back and spinal column undergo elevated stress for a long time [9], chronic back problems are more common than acute incidents [10]. Fatigue of the trunk muscles induced by excessive loading of the spine is one of the sources of back problems in athletes [11]. In particular, high training volume and repetitive trunk motions are responsible for the high prevalence rates [11,12].

Athletes involved in impact sports with repetitive loading of the spine are at risk for certain spinal pathologies [9]. Spondylolysis and degenerative disc diseases are frequent in athletes practicing intense training for a long time [9]. Inflammation around the vertebrae and back muscles often leads to back pain and may even cause disk injuries [1]. Sciatica can occur in cyclists who are in a flexed forward posture or in athletes of water and swing sports who practice large amounts of trunk rotations. Sacroiliac joint dysfunction is a cause of low back pain in athletes who are exposed to repetitive asymmetric loading [13].

Asymmetric loading causes side-to-side disbalance, which may enhance susceptibility to back pain or injury. This assumption may be supported by an association between repeated golf swings and golf-related low back injuries [14]. In golfers, as well as in hockey and tennis players, significantly lower trunk rotational power on the non-dominant than on the dominant side was found (∼15%, 14%, and 12%, respectively) [15]. These values were double compared to non-significant between-side differences in recreationally active individuals practicing or playing sports without such rotational demands (∼7%) [15].

Back problems are also frequent in wheelchair athletes, who suffer from spine curvature disorders [16]. The disability of these athletes participating in Paralympic court sports may impact their sporting performance [17]. For instance, wheelchair table tennis players exhibit lower mobility in the thoracic and lumbar regions of the spine during trunk flexion as well as lower lumbar inversion and pelvic retroversion than able-bodied athletes [18]. Their decreased posterior concavity and limited range of motion during trunk rotations contribute to lower trunk rotational velocity [18].

Besides athletes, low back syndromes are typical for dancers whose spine is greatly affected while performing extreme trunk and lower limb movements to achieve desired positions [19]. For instance, young ballet dancers showed a higher incidence of joint hypermobility, greater mobility of the thoracic spine, a less prominent lordosis of the lumbar spine and a less prominent kyphosis in the thoracic spine in the neutral standing position when compared with the controls [20]. Similarly, the spinal morphology of Latin American-style professional dancers is characterized by lower thoracic kyphosis and lumbar lordosis and anterior pelvic tilt in the standing posture as well as a flexible spine, especially in flexion postures, and suitable hamstring muscle extensibility [21]. Though specific dance postures and movements modify their spinal curvatures, they do not alter the spinal morphology in standing when compared with non-dancers.

Contrary to these studies investigating the role of sport-specific risk factors (e.g., demands on the spine, the training volume, internal and external loads, etc.) in increasing the occurrence of back pain and injuries [3,22,23], less attention has been paid to the investigation of positive effects of sports (e.g., dancing, swimming, tai-chi, yoga, etc.) on prevention and/or reduction of back problems in athletes. A systematic review by Shahtahmassebi [24] identified that the largest effects of exercise on trunk muscle morphology have been reported by studies implementing training programs consisting of motor control exercises combined with non-machine-based and machine-based resistance exercises.

Nevertheless, there is no sufficient evidence to identify sports that could help ease back pain through improvement of core strength, postural stability, and flexibility. A recent systematic review by Gordon and Bloxham [25] revealed that a general exercise program that combines muscular strength, flexibility, and aerobic fitness is beneficial for rehabilitation of non-specific chronic low back pain. Increasing core muscular strength can assist in supporting the lumbar spine [25]. Improving the flexibility of the muscle tendons and ligaments in the back increases the range of motion and assists with the patient’s functional movement [25]. Aerobic exercise increases the blood flow and nutrients to the soft tissues in the back, improving the healing process and reducing stiffness, which can result in back pain [25]. However, the question remains as to whether a positive effect exists on back health following core-specific workouts in competitive athletes within their sports, and if yes, which of these workouts is the most effective. This scoping review aimed at (i) mapping the literature that addresses the effects of core strengthening exercises within the athlete training on reduction of LBP incidence and/or its prevention, (ii) analyzing the relationship between the level of core muscle strength and LBP in athletes of team and individual sports, and (iii) identifying gaps in the existing literature and proposing future research on this topic.

## 2. Materials and Methods

The article was designed as a scoping review [26]. A literature search was conducted to answer the above questions and to identify gaps in existing research. The electronic literature was searched on the MEDLINE, PubMed, Web of Science, Scopus, and Cochrane Library databases. Further searches were conducted on Elsevier, SpringerLink, and Google Scholar. Articles published in peer-reviewed journals and conference proceedings were analyzed. A manual search for references included in research articles and reviews was conducted to identify other relevant studies. If multiple publications included overlapping data resulting from the same study, those with higher numbers of participants and/or the most recent publication dates were analyzed. The literature search was limited to English and German languages.

The search was performed independently by the authors of the study. It was confined to studies closely associated with the major topic of this review, i.e., analyzing the role of athlete training with differing demands on the core musculature in reduction and/or prevention of back problems. Our primary focus was on the positive effects of core-specific workouts used in athletes of individual and team sports on back health. This approach, however, led to the identification of a limited number of studies that were able to meet the eligibility criteria for this review. The search was later widened to include all relevant studies that carried out similar research; however, no significant results were reported. Studies were included if a relationship between the level of core muscle strength and back pain was investigated. This enabled us to identify gaps in the current literature regarding the benefits of sport-specific training loads on the core musculature in decreasing the risk of back problems in athletes and to suggest a proposal for future research.

The target population was competitive athletes of individual and team sports. Keywords referring to “back pain” (“back problems,” “back pain,” and “low back pain”) were combined with keywords referring to “core” or “training” (“athlete training,” “training program,” “core training,” “core muscles,” “core musculature,” “core strength,” and “core stability”) using the Boolean command ‘and’. These keywords were then further combined with sport terms (“basketball,” “canoeing,” “combat sports,” “cricket,” “cycling,” “football,” “golf,” “gymnastics,” “handball,” “hockey,” “power lifting,” “rowing,” “rugby,” “skiing,” “soccer,” “swimming,” “tennis,” “track and field,” “triathlon,” “volleyball,” and “wrestling”) using the Boolean command ‘or’. Additional searches were performed using the words from subheadings, such as exercises contributing to reduction and/or prevention of back problems in athletes.

Studies were excluded if their title did not include at least one keyword of the three basic keyword categories referring to “back pain,” “core,” “athletes” and/or “sports,” and if they were incomplete (abstracts), not peer-reviewed, or did not contain original research. Books, dissertations, theses, and case studies were also excluded. Studies published after the year 2000 were preferred. The key inclusion criteria were as follows: (i) the population of interest consisted of athletes of individual and team sports (youth, adult, national, and international competitions); (ii) a prospective or cross-sectional cohort design was used; (iii) a related prevalence rate of LBP was reported; (iv) the training program in intervention studies was described; (v) a randomized controlled trial (single-blinded or double-blinded) was conducted, and (vi) valid and reliable testing methods were used. Studies that failed to meet the eligibility criteria for this review were excluded.

The process of exclusion and inclusion was conducted independently by both the authors of the study. Different meanings on the inclusion were discussed until the consensus was reached. Qualitative appraisal of selected studies was conducted independently by both authors of the review. Randomized and non-randomized experimental studies were assessed using a modified Downs and Black checklist [27] based on Cochrane risk of bias [28]. In the randomized studies, item Nos. 5, 8, 9, 14, and 26 were excluded, and in the non-randomized studies, item Nos. 5, 7, 8, 9, 14, 15, 23, and 26 were excluded. Studies of at least moderate quality were included. All seven studies corresponded to that. Some concerns were related to sample representativeness, missing follow-ups and non-controlled compliance of the interventions, measures undertaken to address and categorize non-responders, non-measured risk factors, and the missing information about non-responders. Cross-sectional and observational studies were assessed using AXIS [29]. Studies that met at least 11 out of 20 assessed items were included. The score of studies was 11–15 out of 20.

**Figure 1:**
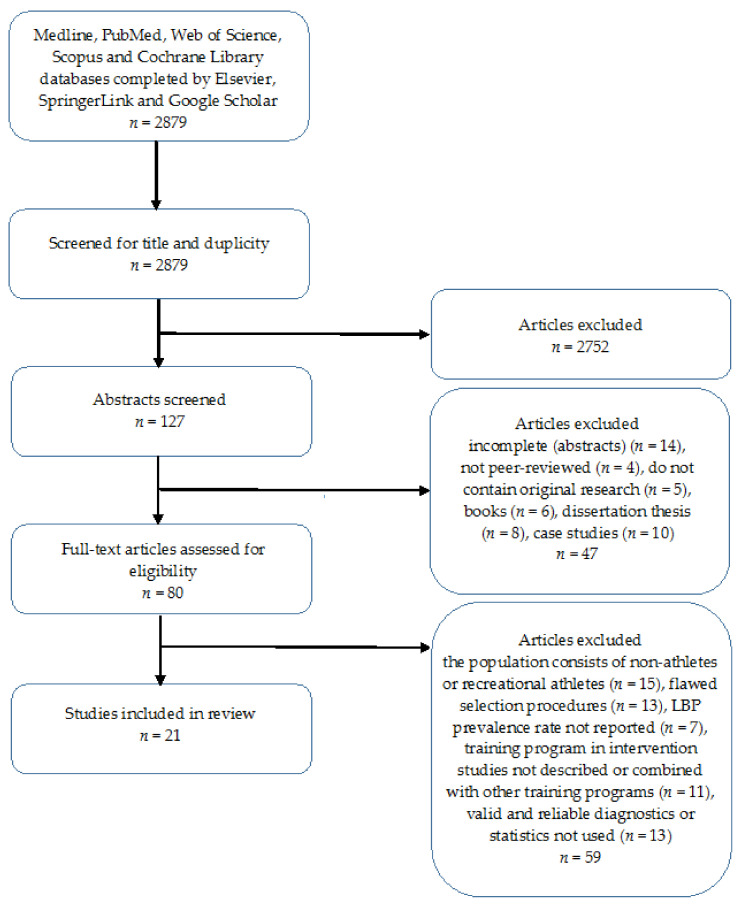
Flow chart of study design.

Particular phases of the search process are shown in Figure 1.

## 3. Results

### 3.1. The Overview of Eligible Studies

Out of the 21 selected studies, fourteen studies (66.7%) [30,31,32,33,34,35,36,37,38,39,40,41,42,43] were aimed at investigating the relationship between LBP and the morphology of the lumbar multifidus muscle (LMM), strength, and strength endurance of core muscles in different sports. The remaining seven studies (33.3%) [44,45,46,47,48,49,50] investigated the effect of different core training programs on the prevention or reduction of LBP in various sports.

Out of 15 studies aimed at investigating the relationship between LBP and the morphology of LMM, strength, and strength endurance of core muscles, 11 (73.3%) had a cross-sectional character [30,31,32,36,37,38,40,41,42,43] and four (26.7%) were prospective studies [33,34,35,39]. Regarding studies that investigated the effect of different core training programs on the prevention or reduction of LBP, five (66.7%) were experimental [45,46,47,49,50] and one was quasi-experimental [48]. Six studies (28.6%) were conducted between 2000 and 2010 [42,43,46,47,48,49] and 15 (71.4%) studies between 2011 and 2020 [30,31,32,33,34,35,36,37,38,39,40,41,44,45,50].

According to the type of sport, 13 studies (61.9%) were related to team sports (soccer, volleyball, handball, basketball, ice hockey, hockey, and cricket) [30,31,32,33,34,35,36,37,44,45,48,49,50] and eight studies (38.1%) were related to individual sports (tennis, golf, rowing, wrestling, gymnastics, and team gymnastics) [38,39,40,41,42,43,46,47].

Approximately one-half, i.e., 11 studies (52.4%) were conducted with elite athletes (young or adult) [33,36,37,38,40,41,42,43,44,47,48]. The remaining 10 studies (47.6%) were conducted with regularly competing university or collegiate athletes or athletes from lower leagues [30,31,32,34,35,39,45,46,49,50]. Seventeen studies (81%) were conducted with adult athletes [30,31,33,34,35,38,39,40,41,42,43,44,45,46,48,49,50] and four studies (19%) with youth athletes [32,36,37,47].

Regarding the gender, twelve studies (57.1%) were conducted with male athletes [30,33,36,37,40,42,43,44,45,48,49,50] and only six studies (28.6%) with female athletes [32,34,35,39,46,47]. Three studies (14.3%) included both male and female athletes [31,38,41].

### 3.2. Relationship between Core Muscle Characteristics and LBP in Cross-Sectional and Prospective Studies

As mentioned above, 14 studies investigated the relationship either between core muscle characteristics and LBP [36,37,40,43] and risk factors of LBP [35], or between neuromuscular control of spine stability and LBP [41]. Among core muscle characteristics, the size, CSA, and contraction or activation (8 studies, 57.1%) [31,33,34,35,36,37,38,41], and, above all, musculus multifidus [31,34,35,37] were the most studied. Four studies (28.6%) were devoted to core endurance [30,38,39,42], and four others (28.6%) to isometric trunk muscle strength [33,40], isokinetic trunk muscle strength, and trunk rotation strength [41,42]. Other studies assessed several different strength parameters, in some cases combined with the morphological characteristics of core muscles [32,36,43].

Regarding the sample, in most studies athletes were divided into a group with LBP and into a group without LBP [30,31,32,33,34,35,36,37,38,39,42]. In two studies, athletes with LBP were compared with healthy counterparts and healthy controls within the general population [40,43]. In another study, athletes with LBP and without LBP were compared with controls within the general population with and without LBP [41].

With regard to the methodology, only special self-administered questionnaires were used to assess LBP in five studies (37.5%) [32,33,34,35,40]. The most widely used standardized test was the Visual Analogue Scale (VAS), often combined with complementary questionnaires, to obtain information about the frequency and date of LBP incidence [31,36,37,41,42]. Others used standardized questionnaires such as the Micheli functional scale (MFS) [30], Nordic Musculoskeletal Questionnaire [38], and Oswestry Disability Questionnaire [43]. The LBP was also evaluated clinically by medical staff [43] and physiotherapists [40].

Core muscle characteristics and related variables (e.g., flexibility, neuromuscular control of spine stability) were diagnosed with ultrasound imaging [31,33,34,35,36,37] and surface electromyography [38]. Moreover, various field tests of maximal strength or strength endurance were used, such as McGill′s endurance test [34], the Swiss Olympic test (SOT) [32], and the Sorensen endurance test [36,37,39]. Under laboratory conditions, core muscle strength was assessed using various isoinertial, isokinetic, or isometric dynamometers, for example, Dynamometer Targumed 700 (Proxomed, Alzenau, Germany) [32], Commander Power Trak II (J-Tech Medical, UT, USA) [33], Dynamometers David 110°, 120°, 130°, and 150° (David International, Ltd., Vantaa, Finland) [40], Dynamometer Biodex 3 (Medical System Inc., USA) [41], Biodex System3 (Biodex Corp., Shirley, NY, USA) with a back attachment [42], and Isokinetic Dynamometer Biodex System III (Biodex Medical System, Inc., Shirley, NY, USA) with a torso rotation attachment [43]. Furthermore, a combination of various strength, balance, flexibility, and quick release tests or radiological evaluation was applied [32,33,36,37,39,40,41,42,43]. Studies dealing with relationships between core characteristics and LBP in team and individual sports are displayed in Table 1 and Table 2. 

### 3.3. Core Muscle Training and LBP

The most used interventions for the prevention or reduction of LBP occurrence were core stability training (CST) with and without virtual reality training (VR) [45], a pre-season core training program [46,49], specialized core trainings [44,47,49], isokinetic training (IKT) and core stabilization training (CST) [50].

The duration of intervention was from 6 to 13 weeks, 2–5 times a week, at least 15 min of each training consisting of core exercises conducted by researchers or coaches [45,46,48], the whole pre-season core training program consisting of self-managed exercises by athletes [44] or conducted by physiotherapists in durations of 4–13 weeks, 5 days per week, 20–40 min per day [47,49,50].

Training programs included core stability exercises (CSE) [48], core stability exercises (CSE) plus virtual reality training (VR) [45], endurance core exercises [46], dynamic stabilization techniques vs. conventional treatment ultrasound, short-wave diathermy, lumbar strengthening [49], specific segmental muscle training based on isometric contractions [47], isokinetic training (Biodex) vs. core stabilization training on Swiss balls and vs. conventional stabilization training [50], and self-managed exercises [44].

Randomized groups were in four studies [45,47,49,50], the population control group was in one study [46], and athletes divided into a group with LBP and without LBP were in two studies [44,48].

LBP diagnostics included mainly Micheli functional scale (MFS) [45], Visual Analogue Scale (VAS) [50], Borg´s category ration scale [47], and special self-administered questionnaires [44,48]. Tracking of back pain was also provided in training by researchers or coaches [46] and clinically by sports medicine staff [49].

Diagnostics of core muscle characteristics and related variables was carried out using ultrasound imaging [44,48,49] and various tests of balance, strength, and flexibility [45,46,50], such as the Star excursion balance test [45], the Biering–Sorensen test [46], and so forth. Studies dealing with core muscle training and LBP are displayed in Table 3.

## 4. Discussion

### 4.1. Significant Relationship between Core Muscle Characteristics and LBP

Poor strength endurance of core muscles and trunk rotation endurance in athletes is most likely associated with LBP [30,33,39,43]. More specifically, negative correlations were reported between the Micheli functional scale and trunk extensor and flexor endurance tests in the group of soccer, basketball, handball, and volleyball players with non-specific LBP [30]. Moreover, asymmetry in hip adductor and abductor muscle strength was found between elite soccer players with and without LBP [33]. Furthermore, rowers with the Functional Movement Screen (FMS) score ≤ 16 had a shorter plank-test hold time, indicating that a lack of core endurance may contribute to the increased risk of LBP [39]. However, golfers with LBP only demonstrated significantly lower endurance in the non-dominant direction (the follow-through of the golf swing) than healthy groups [43].

Furthermore, there was a tendency for higher maximal isometric strength of trunk muscles in soccer players without LBP [32]. However, its values were lower in athletes and non-athletes with LBP than in healthy athletes and controls without LBP. Athletes and controls with LBP developed different strategies to ensure spine stability after perturbation when compared to healthy individuals [41]. Specific deficits in core muscle morphology (thickness, cross-sectional area), activation, and contraction could also be associated with LBP [31,33,34,35,36,38].

### 4.2. Non-Significant Relationship between Core Muscle Characteristics and LBP

The findings revealed no significant difference in trunk rotation endurance between healthy elite golfers and the non-golfing controls [43]. In addition, no significant differences in peak torque were found within or between groups of golfers [43]. Similarly, there were no significant differences in maximal isometric or isokinetic strength of trunk muscles between athletes with and without LBP [40,42]. Furthermore, there were no significant differences between LBP and non-LBP athletes in specific deficits of core muscle morphology [37]. However, these findings were not associated with age, sex, or participant′s level of performance but appeared to be related more to the research methods used.

### 4.3. Core Muscle Training and LBP

Trunk extensor endurance exercises [46], specific segmental muscle training programs [47], stabilization training on multifidus muscle CSA [48], dynamic muscular stabilization techniques [49], and also self-managed exercises by athletes [44] are able to improve the strength or morphological characteristics of core muscles and prevent or reduce LBP. For instance, training on an isokinetic dynamometer (Biodex, Medical System 3, Inc., NY, USA) reduced pain intensity and improved well-being and sports performance more than core stabilization training on a Swiss ball or conventional balance training [50]. Furthermore, core stability exercises combined with virtual reality improved dysfunction levels in non-specific LBP more effectively than core stability exercises alone [45]. Moreover, muscular stabilization techniques were more effective in the treatment of LBP than ultrasound, short-wave diathermy, and lumbar strengthening [49].

To study the effect of core stabilizing and core strengthening exercises on reduction of LBP in young athletes is of special importance. In some sports they are exposed to training with high demands on core musculature that may cause LBP. Children and adolescents with LBP are at higher risk of back problems in adult life than their peers without LBP. Besides sport-specific factors [11], also anthropometric, biomechanical, behavioral, psychological, and genetic factors or common exposure to environmental factors may play a role in high prevalence rates [51,52,53]. While genetic factors have little influence on LBP in children at 11 years [54] and adolescents aged 12–15 [55], symptoms seem to be related to a mixture of shared (41%) and unshared (59%) environmental factors [54]. The shared environment consists of the family environment and shared influences of school, neighborhood, social class, etc., whereas the non-shared environment includes factors that are unique for each family member [54]. Hence, prospective and intervention studies are needed to identify potential environmental and sport-specific factors in order to design the most effective core-specific workouts for the prevention of back problems in adulthood.

### 4.4. Gaps in Current Studies Investigating the Effect of Core Strengthening Exercises within Athlete Training on Back Health and Proposals for Future Research

Analysis identified these gaps in the current literature:

(i) There is only a small number of experimental studies. The proportion of experimental to observational studies (ex post facto) is 28.5%:71.5%.
(ii) In ex post facto experimental design, cross-sectional studies prevail over prospective studies (long-term interventions) in the ratio of 73.3%:26.7%.
(iii) There is a lack of studies in individual sports. In proportion to team sports, it is 38.1%:61.9%.
(iv) There is a lack of studies with young athletes. In proportion to adult athletes, it is 23.8%:76.2%.
(v) There is a lack of studies with female athletes. Though in a small number of studies, data of both female and male athletes were analyzed, these were not compared. The proportion of female, male and both genders is 28.6%:57.1%:14.3.0%.
(vi) Most of the ex post facto studies, 57.1%, were oriented at morphological characteristics of core muscles, a further 35.7% at core strength endurance and only 28.6% at maximal isometric or isokinetic core strength.
(vii) In the diagnostics of LBP, a self-administered questionnaire was used in up to 33.3% of the studies, while a standardized questionnaire complemented with self-administered was applied in 19.1% of the studies.
(viii) Controls represented by the healthy population without marked physical activity participated only in 19% of the studies, and randomized groups occurred only in 19%.
(ix) There is a lack of information as to whether a special core program in experimental studies was or was not combined with a regular training routine, and if yes, what was the training about.
(x) In ex post facto experimental design, particularly in prospective studies, it is not measured whether athletes, usually before the second testing within the frame of their training program, conducted core muscle training or other forms of strength or balance training, their amount, and the type of training or competitive loads.

Based on an analysis of the literature, implications for future research can be formulated as follows:

(i) More experimental rather than observational studies should be conducted.
(ii) Long-term interventions over cross-sectional studies should be preferred.
(iii) Sports that have higher rates of back pain include gymnastics, diving, weight lifting, golf, American football, and rowing [56]. Thus, a higher proportion of studies in individual sports with high demands on core musculature should be conducted.
(iv) Young athletes who present with low back pain have a high incidence of structural injuries such as spondylolysis and other injuries to the posterior elements of the spine [57]. Therefore, more research should be conducted with young athletes to avoid serious problems in older age.
(v) The number of studies conducted with female athletes should be increased. Female athletes are prone to small, hairline fractures of the lumbar spine, usually from overtraining or improper loading of the spine.
(vi) Compared to core strength endurance, maximal isometric and isokinetic core strength, muscle power during trunk rotations and lifting tasks should also be investigated.
(vii) In comparison with frequently used questionnaires, laboratory and field tests of core muscle strength, power, and endurance, spine stability and flexibility should be used to complement the functional testing of athletes prone to back pain and injuries.
(viii) The randomized process and experimental and control groups should be more precisely described.
(ix) Besides the core strengthening and core stabilization exercises, the athlete training (the type, volume, frequency, etc.) should also be specified.
(x) Moreover, information on other forms of strength, balance, or flexibility exercises should be provided.

## 5. Conclusions

Analysis of 21 eligible studies revealed that in 17 of them core strengthening and core stabilization exercises, alone or in combination with athlete training, were effective in improving the strength of core musculature and thereby contributing to the reduction of back pain in athletes. However, no significant association of core muscle strength and/or endurance with indicators of back pain was identified except in four studies. Nonetheless, more research is warranted to elucidate the pros and cons of purely sport-specific training with differing demands on the core musculature on either reducing or developing back problems in athletes. This could help us to design prevention strategies specifically tailored to individual athletes.

## Figures and Tables

**Table 1:** Relationships between core characteristics and LBP in team sports.

Authors	Study Design	Subjects/Sports/Performance Level	Results	Conclusions
Abdelraouf and Abdel-Aziem 2016 [30]	Cross-sectional study	55 collegiate male soccer, basketball, handball, and volleyball players 30 players with LBP, 25 without LBP, age 21.5 ± 2.54 y	Significant differences in endurance tests between athletes without and with LBP Good and moderate negative correlations between MFS and trunk extensor and flexor endurance tests in the group with LBP	Poor core endurance is likely associated with LBP in team players
Fortin et al. 2019 [31]	Cross-sectional study	32 university ice hockey players (18 females, 14 males)	Significantly smaller resting LM CSA and thickness in prone position and higher LM side-to-side asymmetry in standing in players with LBP	Specific deficits in LM morphology in athletes with LBP LM morphology is strongly associated with body composition
Haag et al. 2016 [32]	Cross-sectional study	18 female soccer players U15–U17 competing in the first, second, or third league (8 with and 10 without LBP)	Players without LBP are significantly better in the dynamic lateral isometric muscle strength tests and non-significantly better in the SOT and the YBT	Tendency for better results in strength tests in players without LBP
Hides et al. 2016 [33]	Prospective study	23 elite soccer players at the start and the end of pre-season, age 24.4 ± 5.5 y, playing years 6.4 ± 5.7	28% players reported LBP at the start of the pre-season LBP is associated with reduced size of multifidus muscles, increased contraction of transversus abdominis, and multifidi LBP decrease across the pre-season on improving the size of multifidus There are differences in hip adductor and abductor muscle strength between players with and without LBP	Identified modifiable factors associated with reduced LBP could help to develop more targeted pre-season rehabilitation programs
Myrer et al. 2014 [34]	Two years longitudinal study	12 university female volleyball players, age 19.3 ± 1.3 y	Significant decrease in LM CSA at the 5th vertebral level from pre-season to post-season in all players Significantly smaller LM CSA at the 4th lumbar vertebral level in players with than without LBP	Smaller LMM CSA at the 4th lumbar vertebral level may be the cause of LBP in volleyball players
Nandlall et al. 2020 [35]	Prospective study	11 female and 7 male university soccer players	Significantly higher LM % thickness change during contraction in the prone position in players with LBP LM CSA and echo intensity positively correlated with total body fat	Grater LM contraction in players with LBP is probably a maladaptive strategy to splint and project the spine Decrease in LM thickness is associated with lower-limb injuries during playing season
Noormohammadpour et al. 2019 [36]	Cross-sectional study	30 male premier soccer league players, age 17.4 ± 1.1 y, training week hours 9.8 ± 1.7	Significantly lower external oblique muscle thickness bilaterally and internal oblique muscle thickness on both sides, and lower hamstring flexibility in subjects with a sport life history of LBP than in the non-LBP group on the dominant limb	Lower external and internal oblique muscle thickness bilaterally may be a risk factor of sport life history of LBP
Noormohammadpour et al. 2016 [37]	Cross-sectional study	28 premier soccer league players LBP group: *n* = 14, age 14.0 ± 1.1 y Non-LBP group: *n* = 14, 14.1 ± 0.9 y Training hour/week: LBP group 5.8 ± 0.8 h, non-LBP group 5.9 ± 1.8 h	Non-significant differences between LBP and non-LBP groups in abdominal muscle thickness and CSA of LMM, hamstring tightness, leg length discrepancy, isometric muscle endurance of trunk extensors, and active lumbar forward flexion	No relationship between abdominal muscle thickness and CSA of the lumbar multifidi and LBP in adolescent soccer players

LBP-Low Back Pain, LM-Lumbar Multifidus, LMM-Lumbar Multifidus Muscle, CSA-Cross Sectional Area, SOT-Swiss Olympic Test, YBT-Y Balance Test, y-Years, MF-Median Frequency, h-Hour.

**Table 2:** Relationships between core characteristics and LBP in individual sports.

Authors	Study Design	Subjects/Sports/Performance Level	Results	Conclusions
Correia et al. 2016 [38]	Cross-sectional observation study	28 male and 7 female tennis players (15 without LBP), age 18.54 ± 3 y, min. 3 y of playing practice, 6 h weekly, national and higher level	Players with LBP: reduced activation of left and right ES-I and left longissimus thoracic in extension test, lower average EMG slope of left EO and left RA MF slope in the left side bridge, lower left ES-I average EMG slope in the right-side bridge Players without LBP: better flexor and right-side bridge times, greater increase in average EMG in the right-side bridge test for left ES-I	LBP in symptomatic players is associated with lower activation of extensor muscles and less with contraction patterns and abdominal endurance
Gonzales et al. 2018 [39]	Prospective cohort study	31 NCAA Division I, female open-weight rowers, age 19.9 ± 1.4 y, rowing experience 5.2 ± 2.5 y	Rowers with FMS score ≤16 have a shorter plank-test hold time and 1.4 times higher risk of developing LBP than those with a score >16 No differences in FMS between LBP and non-LBP groups	Lower core endurance may be a risk factor of LBP in female rowers with an FMS score ≤16 FMS is not recommended for widespread screening of female rowers because of a relatively small risk ratio and its wide 95% confidence interval
Grosdent et al. 2015 [40]	Cross-sectional study	Elite male tennis players with current LBP (*n* = 11, age 27.8 ± 5.5 y) without LBP (*n* = 27, age 24.3 ± 5.9 y) Controls (male students, age 24.8 ± 4.0 y)	Significantly higher strength of lateral flexors and rotators on non-dominant side in tennis players Non-significant differences in trunk muscle strength and ratio of lumbar spine flexibility between players with and without LBP	There are no differences in trunk strength and flexibility in tennis players with and without LBP
Moreno Catalá et al. 2018 [41]	Cross-sectional study	Soccer, handball, judo, gymnastics, and track and field athletes (*n* = 30) and controls (*n* = 29) with or without LBP, age 19–30 y (male *n* = 39, female *n* = 20), training at least 4 times/week, regular participation in national or international competitions	Lower maximal trunk extension moments, higher trunk damping and shorter onset times of trunk muscles in LBP athletes and non-athletes than in healthy individuals Non-significant differences in trunk stiffness and local dynamic stability between LBP athletes and controls and healthy controls	There is the same activity of lumbar extensor muscles and similar strategies to ensure spine stability after a sudden perturbation in athletes and controls with LBP
Iway et al. 2004 [42]	Cross-sectional study	Collegiate male elite wrestlers (*n* = 53), 35 with and 18 without radiological abnormalities, age 19.5 ± 1.2 y, wrestling history 5.3 ± 3.2 y, 4 h wrestling practice daily, 6 times a week	Peak torque at 120°·s^−1^, work at 60°·s^−1^ and 90°·s^−1^, and average torque at 90°·s^−1^ and 120°·s^−1^ correlate with the LBP indicated in questionnaires None of the trunk flexor parameters significantly correlate with the disability level of LBP	The relatively low isokinetic strength of trunk extensors may be a factor related to non-specific chronic LBP in collegiate wrestlers
Lindsay et al. 2006 [43]	Cross-sectional study	32 healthy elite golfers, age 30.0 ± 6.0 y 7 elite golfers with LBP, age 33.3 ± 9.6 y 40 control healthy individuals, age 27.9 ± 4.8 y	Significantly lower endurance in the non-dominant side in golfers with than without LBP Significant differences between groups in non-dominant but not in dominant rotation Non-significant differences in peak torque between groups	Trunk rotation endurance in golfers is more important than strength alone in the prevention of LBP

LBP-Low Back Pain, LM-Lumbar Multifidus, LMM-Lumbar Multifidus Muscle, CSA-Cross Sectional Area, SOT-Swiss Olympic Test, YBT-Y Balance Test, y-Years, ES-I-Iliocostalis Lumborum, EO-External Oblique, EMG-Electromyography, RA-Rectus Abdominis, MF-Median Frequency, NCAA-National Collegiate Athletic Association, FMS-Functional Movement Screen, °-Degree, s-Second, min.-Minute, h-Hour.

**Table 3:** Core muscle training and LBP.

Authors	Study Design	Subjects/Sports/Performance Level	Results	Conclusions
Hides et al. 2017 [44]	Cross-sectional study	242 players of Australian Football League (AFL), age 18–40 y	Significant interaction between MF muscle CSA and MF muscle training over time at the L5 vertebral level but not at the L4 level Significant interaction among MF muscle size, LBP, and MF muscle training over time for the L5 vertebral level but not for the L4 level No interaction among MF muscle CSA, LBP, and pre-season fitness and strength training over time for either the L5 vertebral level or the L4 level	Self-managed exercises are effective at negative changes in the MF muscle across the pre-season Supplementary program targeted on the MF muscle is effective at maintaining MF muscle size in elite AFL players with or without LBP
Abdelraouf et al. 2020 [45]	Randomized clinical trial	55 soccer players with LBP Experimental group (EG): age 20.86 ± 5.17 y Control group (CG): age 22.14 ± 2.58 y	Significantly higher post-values in the EG than the CG for the dynamic balance in anterior, posterolateral, and posteromedial directions Significant difference in the reducing Micheli functional scale in favor of EG	CSE training plus VR is more effective than CSE training alone in improving body balance and dysfunction level in non-specific LBP
Durall et al. 2009 [46]	Experimental study	Experimental group (EG): 15 collegiate women gymnasts, age 19.5 ± 0.3 y Control group (CG): 15 non-athletes, collegiate students, age 19.7 ± 0.4 y	Significant improvements in all core tests in the EG, and non-significant changes in the CG Significantly higher endurance improvements in the EG in all tests, except for one gymnast with chronic LBP No reports of LBP during competitive season	Training program for trunk musculature with relatively simple floor exercises is effective stimulus to improve the endurance of trunk muscles and prevent the occurrence of LBP
Harringe et al. 2007 [47]	Prospective controlled intervention study	Three teams of top-level youth team gymnasts, age 11–16 y Intervention group (IG): two teams, *n* = 33 Control group (CG): one team, n = 18	A significantly smaller number of days with LBP at the end than start of the study in the IG No difference in days with LBP or intensity of LBP in the CG Eight gymnasts out of 15 with LBP in the IG became pain free	Specific segmental muscle control exercises of lumbar spine may prevent and reduce LBP in young gymnasts
Hides et al. 2008 [48]	Single-blinded, pre-treatment– post-treatment study	26 male elite cricketers LBP group: *n* = 10, age 21.4 ± 2.0 y No-pain group: *n* = 16, age 21.9 ± 2.5 y	Increase in CSA of multifidus muscles at the L5 vertebral level in 7 players with stabilization training compared to 14 players without LBP Significant decrease in the amount of asymmetry among players with LBP 50% decrease in the reported pain level at L2–L4 among players with LBP	Specific retraining can improve the multifidus muscle CSA and decrease the pain
Kumar et al. 2009 [49]	Prospective study	30 male hockey players EG: *n* = 15, age 24.07 ± 2.89 y CG: *n* = 15, age 23.4 ± 3.27 y	Dynamic muscular stabilization techniques (DMST) are more effective than conventional treatment in rehabilitation of LBP Greater improvements in the walking, stand ups, climbing, and pain with DMST than conventional treatment	DMST is more suitable than conventional treatment for the management of LBP
Nambi et al. 2020 [50]	Randomized, double-blinded controlled study	60 university male football players with chronic LBP for more than 3 months and 4–8 pain intensity in VAS divided into IKT (*n* = 20), CST (*n* = 20) and CG (*n* = 20), age 18–25 y	Significant differences between IKT, CST, and CG after 4 weeks of training More pain reduction and player′s wellness improvement in the IKT than CST and CG Significant improvement in sports performance variables in the IKT than CST and CG	Training through IKT reduces pain intensity and improves well-being and sports performance more than CST in soccer players with chronic LBP

L-Vertebral Level, CSA-Cross Sectional Area, MF-Median Frequency, IKT-Isokinetic Training, CST-Cross Stabilisation Training, VAS-Visual Analog Scale.
